# Real-time oxide evolution of copper protected by graphene and boron nitride barriers

**DOI:** 10.1038/srep39770

**Published:** 2017-01-09

**Authors:** M. Galbiati, A. C. Stoot, D. M. A. Mackenzie, P. Bøggild, L. Camilli

**Affiliations:** 1 Department of Micro- and Nanotechnology, DK-2800 Kgs. Lyngby, Denmark

## Abstract

Applying protective or barrier layers to isolate a target item from the environment is a common approach to prevent or delay its degradation. The impermeability of two-dimensional materials such as graphene and hexagonal boron nitride (hBN) has generated a great deal of interest in corrosion and material science. Owing to their different electronic properties (graphene is a semimetal, whereas hBN is a wide-bandgap insulator), their protection behaviour is distinctly different. Here we investigate the performance of graphene and hBN as barrier coatings applied on copper substrates through a *real-time* study in two different oxidative conditions. Our findings show that the evolution of the copper oxidation is remarkably different for the two coating materials.

Metals and alloys are used worldwide in nearly all forms of industries. Corrosion – *i.e.*, gradually degradation of metals due to chemical or electrochemical reactions with the environment – causes waste of valuable resources, loss or contamination of products, reduction in efficiency and costly maintenance. Furthermore, failure of critical metal parts is not just expensive, but potentially dangerous. It is therefore one of the most important technological challenges of material science to discover efficient materials and approaches for the prevention of metal corrosion.

Over the last few years it has been suggested to use graphene (G) as a barrier coating to protect metals from oxidation and, more generally, corrosion^1^,^2^,^3^,^4^,^5^. Defect-free graphene is highly impermeable to liquids and gases and inert to most chemicals^6^. Moreover, owing to its one-atom thickness, coatings made of single or even few layers of graphene would not affect the morphology or the appearance of the metal to be coated. However, its electrochemical nobility and the high electronic conductivity cause graphene to form a galvanic cell when directly in contact with a metal substrate and this, eventually, enhances degradation of the substrate in the long term^7^,^8^.

Hexagonal boron nitride (hBN) has recently been proposed as an alternative^9^,^10^,^11^. In addition to having mechanical and impermeable properties comparable to graphene^12^,^13^, hBN is an insulator^14^ and does not induce galvanic corrosion when in contact with metals^9^,^11^.

Since the landmark paper by Li *et al*. in 2009^15^, copper has been by far the most widespread substrate for growth of graphene through chemical vapour deposition (CVD). Indeed, the low solubility of carbon in copper allows the growth process to be self-limiting^15^, which, in turn, allows for a precise control of the number of graphene layers. Copper is also widely used as a growth substrate for hBN^16^,^17^,^18^,^19^. Therefore, while copper is used in many applications, including electronics, the main motivation for basing this study on copper films is the support of high quality, single-layer growth of continuous sheets of both graphene and hBN, which allows for a direct comparison of their barrier properties.

When copper is exposed to air, three different oxides can be formed^20^,^21^,^22^:

- Cu_2_O (cuprous oxide) is the native oxide, generally the one formed first upon reaction with oxygen^23^:
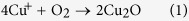
- The metastable Cu(OH)_2_ (copper hydroxide) is formed through the reaction:
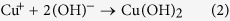
- This metastable layer can then transform into the more stable CuO (cupric oxide) through the reaction:

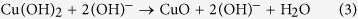


Here, we have investigated the oxidation process in two different conditions, of bare, G-coated and hBN-coated copper through *real-time* Raman spectroscopy, X-ray photoemission spectroscopy (XPS) and X-ray induced Auger electron spectroscopy (XAES). In the first experiment, the specimens were heated from room temperature (RT) up to 400 °C within 45 minutes to simulate a relatively short but acute oxidative condition. The second was an isothermal experiment, where samples were held at 50 °C for 60 h to simulate longer-term oxidative conditions.

## Results

### *Real-time* Raman spectroscopy investigations

#### Variable temperature experiment

**Figure 1:**
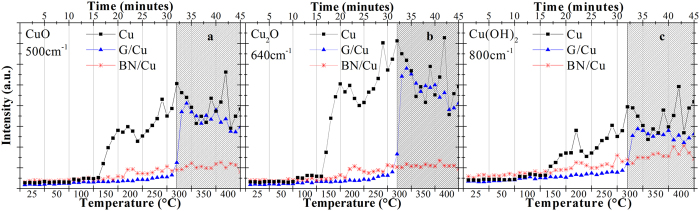
Evolution of the Raman signal intensity as bare (black squares), G-coated (blues triangles) and hBN-coated (red asterisks) copper samples are heated up from RT to 400 °C within 45 minutes for (**a**) CuO at 500 cm^−1^ 3,11 (**b**) Cu_2_O at 640 cm^−1^ 3,11,24,25 and (**c**) Cu(OH)_2_ at 800 cm^−1^ 3,24. The grey area indicates when graphene is etched away upon reaction with ambient oxygen, thus leaving the copper surface uncoated (see Fig. S8 for more details). The vertical lines indicate where the temperature is ramped up, while being kept constant in between.

In the first experiment – called variable temperature (T) experiment - the samples were heated from 25 °C up to 400 °C in steps of 50 °C (only the first step is different, from 25 °C to 50 °C) while the Raman spectra were continuously collected from the same spot on the surface. Figure 1 shows the time evolution of the intensities of the Raman signal for CuO (at 500 cm^−1^ 3,11) and Cu_2_O (at 640 cm^−1^ 3,11,24,25), Cu(OH)_2_ (at 800 cm^−1^ 3,24). In the case of the bare copper sample, both Cu_2_O and, to a smaller extent, Cu(OH)_2_ start forming at around 100 °C (that is, after 10 minutes from the beginning of the experiment; Fig. 1b,c and Fig. S11 in Supplementary Information). In analogy with some previous reports^23^, CuO is instead formed later, after 16 minutes from the beginning of the experiment, which corresponds to a temperature of 150 °C (Fig. 1a). At this point, the overall oxidation of copper proceeds very quickly. The change in Raman response of the sample observed at 150 °C can be due to a change in the oxidation rate. O’Reilly *et al*., in fact, showed that at 150 °C the Cu oxidation rate law switches from inverse-logarithmic to linear^26^.

Regarding the G-coated sample, the Raman intensity signal increases slowly up to a temperature of 250 °C, where the increase becomes more pronounced as indicated by the small change in slope in the relative curves in Fig. 1. Nonetheless, it is only at 300 °C (after 32 minutes from the beginning of the experiment) that a dramatic increase of all the oxide peak intensities is finally observed. Such increase is a consequence of the oxidation of graphene which, once being etched away upon reacting with the atmospheric oxygen^27^ (see Fig. S7 in Supplementary Information), leaves the copper surface completely unprotected. Indeed, at this point, the oxidation level (*i.e.*, the Raman intensities of the individual copper oxide peaks) of the G-coated sample is comparable to that one of the bare sample and, for both samples, the oxide peak intensity increased by a factor of 10 with respect to the initial value.

In the case of hBN-coated copper, the Raman intensity of all the oxide peaks increased very slowly up to a temperature of more than 150 °C (see Fig. S8 in Supplementary Information). At that point, the signal of both Cu(OH)_2_ and Cu_2_O started to increase abruptly. As in the case of bare copper, the CuO Raman signal increased at a significantly higher temperature than the other compounds, at around 200 °C (Fig. S12 in Supplementary Information). At the end of the experiment, the overall degree of oxidation was significantly lower than both G-coated and unprotected sample (Fig. 1).

#### Isothermal experiment

**Figure 2:**
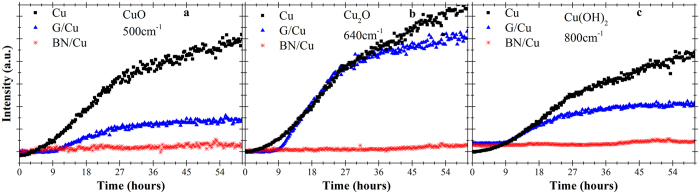
Raman data collected in the isothermal experiment at 50 °C showing the time dependent evolution of the peaks for (**a**) CuO at 500 cm^−1^ 3,11 (**b**) Cu_2_O at 640 cm^−1^ 3,11,24,25 and **(c)** Cu(OH)_2_ at 800 cm^−1^ 3,24.

Figure 2 displays the outcomes of the second *real-time* Raman investigation, *i.e.*, the evolution of the copper oxide Raman peaks while the samples are kept at 50 °C over 60 h. In the bare copper sample, the Raman peak intensities for all the oxide species increase monotonically over the first 24 hours of experiment. After this point, the Raman intensities continue increasing, but at a slower rate.

Interestingly, the G-coated sample shows an initial plateau, which highlights the good barrier performance of graphene upon short-term^7^. 24 hours after the beginning of the experiment, the Raman intensities of all the oxide peaks begin to saturate, similar to the case of the bare copper sample. For G-coated sample at the end of the experiment, the Raman intensity of Cu_2_O and Cu(OH)_2_ was 70% and the CuO was 30% of the bare copper level.

The Raman intensity for hBN-coated copper is almost flat, with hardly any detectable increase over approximately 40 hours. Beyond this point, only the Cu(OH)_2_ peak is increasing. A slight but constant increase in the Cu_2_O is also observable after approximately 45 hours from the beginning of the experiment.

### XPS and XAES studies

**Figure 3:**
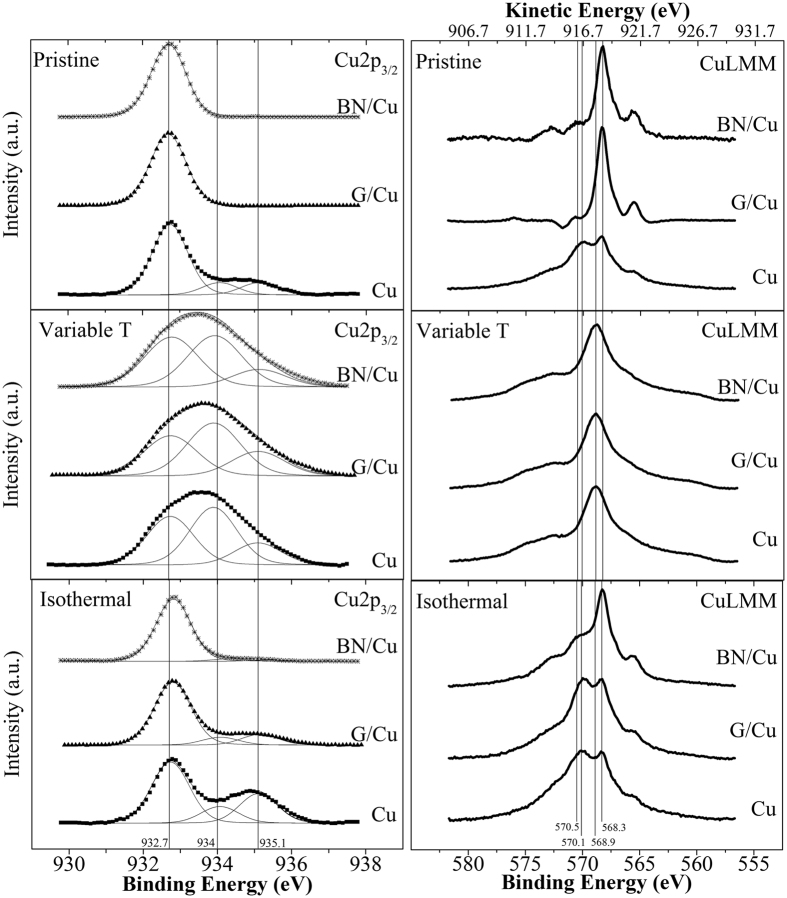
Cu2p_3/2_ core level (left side) and Cu LMM lines (right side) before and after the oxidation experiments for bare, G-coated and hBN-coated Cu samples. In the Cu2p_3/2_ spectra the energy positions for Cu + Cu_2_O, CuO, and Cu(OH)_2_ components are 932.7 eV, 934 eV and 935.1 eV, respectively. In the Cu LMM panel the vertical lines point out the binding energy positions for metallic Cu (568.3 eV)^28^, CuO (568.9 eV)^28^, Cu_2_O (570.1 eV)^28^,^29^ and Cu(OH)_2_ (570.5 eV)^29^. The top horizontal axis in the Cu LMM panels reports the corresponding kinetic energy.

Figure 3 reports the Cu 2p_3/2_ core level and Cu LMM Auger lines for all the tested samples, before (top panel) and after (centre and bottom panels) the two oxidation experiments. As the 2p_3/2_ peaks of metal Cu and Cu_2_O differ by only 0.1 eV, which is below the experimental resolution of the used equipment, we have performed also XAES in order to distinguish between the two components Indeed, as reported in previous studies, the LMM Auger lines are more sensitive than the 2p_3/2_ core level to detect the oxidation of copper^17^.

The 2p_3/2_ peak of the bare copper before the oxidation experiment exhibits three components: one centred at 932.7 eV corresponding to Cu + Cu_2_O, one at 934 eV for CuO and the last one at 935.1 eV for Cu(OH)_2_^20^,^28^,^29^ (Fig. 3, top left panel). Regarding the G- and hBN-coated samples, the Cu 2p_3/2_ core level can be fitted by only one component at 932,7 eV. The XAES data for all the samples before any oxidation experiment are displayed in the top right panel of Fig. 3. The shape of the Auger features is similar for G- and hBN-coated samples and, in both the samples, the intense peak at 568.3 eV suggests that the copper mainly has a metallic character^28^,^29^. In the case of bare copper, the shape of the Auger peak is different, with a similar maximum of the intensity curve for the feature at 568.3 eV (Cu metallic) and for the one at slightly higher binding energy (570.1 eV), this implying the presence of other chemical states rather than metallic Cu on the surface, such as Cu_2_O^28^,^29^.

After the experiment where the temperature was increased to 400 °C (*i.e.*, the variable T experiment), all the samples appeared uniformly oxidized by optical inspection. This is confirmed by the XPS measurements reported in the centre left panel in Fig. 3. The XPS measurements also revealed the absence of boron and nitrogen at the surface after the experiment, while the hBN coating was still present at 300 °C (Fig. S9 in Supplementary Information). A quantitative analysis obtained by peak deconvolution is reported in Table 1. The corresponding XAES measurements for all the samples show typical spectra of CuO surface, peaked at 568.9eV^28^.

The XPS measurements after the isothermal experiment revealed that the coated and the uncoated samples underwent oxidation, although at a different extent. The coated samples, for instance, were significantly less oxidized, as shown by the deconvolution analysis in Table 2. The change in shape of the Auger spectra with respect to the pristine sample for bare and G-coated copper (Fig. 3) confirm that oxidation occurred at the surface, as the two features related to Cu metallic and Cu_2_O, respectively at 568.3 eV and 570.1eV^29^, now have a similar maximum of the intensity curve. The small degree of oxidation for the hBN-coated copper sample is corroborated by the fact that shape of the relative Auger spectrum is qualitatively similar to that of metallic copper, as shown by the higher intensity of the component at 568.3 eV (Fig. 3).

## Discussion

In short (less than one hour) and acute oxidative conditions (*i.e.*, the variable T experiment), graphene acts as an effective oxidation barrier even at temperature as high as 250 °C. This is demonstrated by the fact that the Raman signals of the oxide peaks do not increase significantly until the graphene is etched away upon reaction with the ambient oxygen (Fig. 1 and S7 Supplementary Information). In the temperature range of 150–300 °C, the hBN barrier layer is less effective than graphene at preventing Cu oxides from forming (Fig. 1). This is most likely due to the higher density of grain boundaries (*i.e.*, smaller grains) and wrinkles of the hBN layer with respect to the graphene one (Supplementary Information). It is well known for graphene that grain boundaries, wrinkles and rips have enhanced reactivity and therefore are the points where corrosion and oxidation starts^24^,^30^; we therefore propose to extend this notion to hBN^31^. Therefore, since the activation barrier for oxygen diffusion through hBN and graphene is similar^11^, it is reasonable to assume that the larger density of defects for the hBN coating will induce a faster oxidation of the copper underneath. This is a typical scenario: it is currently much easier to grow high quality graphene than high quality hBN by CVD on copper, as demonstrated by the synthesis of single-crystal graphene of millimetre- or centimetre-size^32^,^33^,^34^,^35^, whereas single-crystal hBN size is still limited to tens of micrometres^18^,^19^. Thus, the polycrystalline hBN synthesized through CVD exhibits many grain boundaries and therefore a relatively high density of structural defects^36^. Above 300 °C, the oxidation rate of G-coated sample increases, and the Raman intensity of all the three copper oxide peaks become relatively larger than the corresponding oxide peaks in the hBN-coated sample, within a very short time. This sudden increase is due to the etching of graphene, as mentioned above, which happens between 32 mins and 33 mins in the temperature range corresponding to 300 °C (Fig. S7 in Supplementary Information). Here, it is worth pointing out that, although also hBN starts to react with the atmospheric oxygen at a temperature around 350 °C (see Fig. S9 Supplementary Information), a sudden increase in the oxide formation is not observed. We ascribe this phenomenon to the absence of a galvanic corrosion component in the case of the electrically insulating hBN^9^,^11^, although also other factors may contribute.

After careful examination of the XPS data, we notice that the surface concentration of Cu^2+^ (*i.e.*, the CuO component) as measured by XPS leads us to conclude that after being heated to 400 °C the samples formed mainly cupric oxides, but the data obtained from the Raman spectroscopy showed that the Cu^+^ (*i.e.*, Cu_2_O) signal at 640 cm^−1^ increased to a greater extent than the cupric oxide signal at 500 cm^−1^ (Fig. 1). We attribute this apparent inconsistency in our data to different penetration depths of Raman spectroscopy and XPS. The laser used for micro-Raman spectroscopy can probe the Cu sample much deeper (tens to hundreds of nanometers) than XPS, which can penetrate only up to few nanometres ~2 nm). Thus the response from Raman spectroscopy included signals coming from regions deeper within the sample, where the oxide composition was different from that of the surface. Both for the graphene-coated and the hBN-coated copper samples after the variable T experiment we find an inner Cu_2_O layer and an outer CuO layer (Figs S15 and S16 in Supplementary Information), in agreement with previous reports in literature^21^,^22^,^37^.

The isothermal experiment highlights the remarkable barrier properties of graphene on short-term, as indicated by the initial plateau in the Raman intensity of the oxide peaks in Fig. 2. After 9 hours at 50 °C, however, graphene coatings failed. The oxidation started resulting in the intensities of the copper oxide peaks increasing rapidly over the next 15 hours with a higher rate than that of the bare copper, with this acceleration being attributed to galvanic corrosion in ambient conditions^8^. After the beginning of the oxidation, the G and 2D Raman peaks are still observed, confirming the presence of graphene. Thus, we could argue that, at first, oxygen did not pass through defects within the individual graphene grains, but rather from existing grain boundaries and wrinkles^24^,^30^. However, after 12 hours, a D peak appears, pinpointing the beginning of the formation of defects within the graphene layer. The defects could be caused by mechanical stresses due to modification of the copper crystal structure induced by its oxidation (Fig. S14 Supplementary Information). Similar results were observed when studying G-coated copper foils left in air at RT for several months^8^.

On the other hand, the hBN layer provides a more effective protection, and even the outmost surface layers of copper are hardly oxidized after 60 hours at 50 °C, as shown by XPS and XAES measurements in Fig. 3, bottom panel. The hBN-coated sample showed a detectable increase for the Cu(OH)_2_ signal after 40 hours; however, the intensity was insignificant in comparison with the other samples, which is strong evidence that hBN is a superior oxygen barrier compared with graphene on the timescale of several hours^11^. In agreement with the Raman data, the combination of XPS and XAES results after the isothermal experiments revealed the Cu_2_O as main oxide component at the surface for both bare and graphene-coated copper. In contrast, the hBN-coated sample showed mainly the metallic copper peak, thus confirming the superior performance of hBN in comparison to graphene as a protective coating against oxidation in long tests.

## Conclusion

In this work we present the first comparative *real-time* study showing the evolution of oxidation of copper samples coated by either graphene or hBN single layers. Raman spectroscopy was used for a *real-time* investigation, while XPS and XAES were used for probing the chemical state of the copper surface before and after the oxidation experiments. Two experiments were carried out: a variable temperature and an isothermal experiment. The former corresponds to a short but acute oxidative condition, while the second simulates a milder but prolonged condition. Both G and hBN coatings provide good protection against Cu oxidation in the short-term (30 minutes) for temperatures below 250 °C, with the graphene coatings outperforming the hBN probably due to its intrinsically higher structural quality (*i.e.*, larger grains and less wrinkles). Raman data for G-coated Cu samples showed no significant increase of any copper oxide peaks in this temperature range. However, in a prolonged oxidative condition, after an initial period of time with no detectable increase in intensity of oxide signals, G failed as protective layer after only a few hours. Due to its electrically insulating nature, hBN lacks the galvanic corrosion component so that even upon longer timeframes copper oxidation is indeed inhibited. We conclude that depending on the application (*i.e.*, the oxidative condition), G coatings or hBN can be chosen. Our results indicate that, when it comes to applications requiring a prolonged protection, hBN coatings will eventually outperform G ones, even if the intrinsic quality of G layer is higher than that of the hBN, which we explain by hBN not supporting galvanic corrosion.

## Methods

The graphene samples were grown using a commercial Annealsys AS-ONE cold-wall chemical vapour deposition (CVD) reactor on 25 μm-thick electropolished copper foils. First, the Cu foils were annealed in argon at 1035 °C in atmospheric pressure for 10 minutes, then graphene was synthesised at the same temperature at a pressure of 25 mbar using a mixture of 900sccm Ar, 60sccm H_2_ and 2sccm CH_4_ for 15 minutes. hBN coatings were grown using a quartz tube furnace on the same batch of electropolished copper foils used for growing graphene. The growth was done at 900 °C at a total pressure of 60 mbar for 15 minutes with a mixture of 300sccm Ar, 15sccm H_2_ and 3sccm of borazine (B_3_H_6_N_3,_ from Fluorochem).

The oxidation experiments were performed in air, with 35–45% of relative humidity. Before the oxidation experiments, the bare copper samples were annealed in Ar at 1035 °C for 15 minutes. Raman measurements were carried out using a Thermo Scientific DXR confocal Raman microscope with a 10× objective and an excitation wavelength of 455 nm using a Linkam LN600P heating stage. In the isothermal experiment the samples were kept at 50 °C for 60 hour meanwhile Raman spectra were collected every 10 minutes using a collection time of 3 minutes per spectrum. All the spectra from a sample were collected from the same spot on the surface. For the variable temperature experiment, a step-ramp of temperature was used: the first step was from 25 to 50 °C and afterwards the temperature was rapidly increased by 50 °C for each step. All the intervals were kept constant in temperature for 5 minutes (as highlighted by the vertical lines in Fig. 1) and the Raman spectra were collected every minute, using a collection time of 1 minute per spectrum. The background was subtracted from all the Raman spectra and then each spectrum was normalised to the nitrogen peak (2329 cm^−1^). XPS and XAES data were collected using a Thermo Scientific Al K-alpha X-ray Photoelectron Spectrometer (1486.7 eV). Shirley background was removed to XAES data. After background subtraction, the Cu 2p_3/2_ peaks were fitted using Voigt functions with full width half maximum of 0.15–0.22 eV and 1–1.5 eV for the Lorentzian and Gaussian component, respectively.

## Additional Information

**How to cite this article**: Galbiati, M. *et al*. Real-time oxide evolution of copper protected by graphene and boron nitride barriers. *Sci. Rep.* **7**, 39770; doi: 10.1038/srep39770 (2017).

**Publisher's note:** Springer Nature remains neutral with regard to jurisdictional claims in published maps and institutional affiliations.

## Supplementary Material

Supplementary Information

## Figures and Tables

**Table 1:** Quantitative analysis of the surface composition of the bare, G-coated and hBN-coated copper samples after the experiment up to 400 °C, resulting from deconvolution of XPS data in Fig. 3 centre left panel.

Variable T experiment	Cu + Cu_2_O (%)	CuO (%)	Cu(OH)_2_ (%)
Cu	38	45	17
G/Cu	41	44	15
BN/Cu	42	43	15

The experimental error is approximately 1%.

**Table 2:** Quantitative analysis of the surface composition of the bare, G-coated and hBN-coated copper samples after the isothermal experiment at 50 °C for 60 hours, resulting from deconvolution of XPS data in Fig. 3 bottom left panel.

Isothermal	Cu + Cu_2_O (%)	CuO (%)	Cu(OH)_2_ (%)
Cu	59	19	22
G/Cu	77	9	13
BN/Cu	94	4	2

The experimental error is approximately 1%.

## References

[b1] Prasai D., Tuberquia J. C., Harl R. R., Jennings G. K. & Bolotin K. I. Graphene: Corrosion-inhibiting coating. ACS Nano 6, 1102–1108 (2012). 22299572 10.1021/nn203507y

[b2] Weatherup R. S. . Long-Term Passivation of Strongly Interacting Metals with Single-Layer Graphene. J. Am. Chem. Soc. 137, 14358–14366 (2015). 26499041 10.1021/jacs.5b08729 PMC4682849

[b3] Chen S. . Oxidation resistance of graphene-coated Cu and Cu/Ni alloy. ACS Nano 5, 1321–1327 (2011). 21275384 10.1021/nn103028d

[b4] Kirkland N. T., Schiller T., Medhekar N. & Birbilis N. Exploring graphene as a corrosion protection barrier. Corros. Sci. 56, 1–4 (2012).

[b5] Stoot A. C., Camilli L., Spiegelhauer S.-A., Yu F. & Bøggild P. Multilayer graphene for long-term corrosion protection of stainless steel bipolar plates for polymer electrolyte membrane fuel cell. J. Power Sources 293, 846–851 (2015).

[b6] Bunch J. S. . Impermeable atomic membranes from graphene sheets. Nano Lett. 8, 2458–2462 (2008). 18630972 10.1021/nl801457b

[b7] Schriver M. . Graphene as a long-term metal oxidation barrier: Worse than nothing. ACS Nano 7, 5763–5768 (2013). 23755733 10.1021/nn4014356

[b8] Zhou F., Li Z., Shenoy G. J., Li L. & Liu H. Enhanced Room Temperature Corrosion of Copper in the Presence of Graphene. ACS Nano 7, 6939–6947 (2013). 23883292 10.1021/nn402150t

[b9] Li L. H., Xing T., Chen Y. & Jones R. Boron Nitride Nanosheets for Metal Protection. Adv. Mater. Interfaces 1, 1–6 (2014).

[b10] Liu Z. . Ultrathin high-temperature oxidation-resistant coatings of hexagonal boron nitride. Nat. Commun. 4, 2541 (2013). 24092019 10.1038/ncomms3541

[b11] Shen L. . A long-term corrosion barrier with an insulating boron nitride monolayer. J. Mater. Chem. A 4, 5044–5050 (2016).

[b12] Song L. . Large scale growth and characterization of atomic hexagonal boron nitride layers. Nano Lett. 10, 3209–3215 (2010). 20698639 10.1021/nl1022139

[b13] Lindsay L. & Broido D. a. Enhanced thermal conductivity and isotope effect in single-layer hexagonal boron nitride. Phys. Rev. B 84, 1–6 (2011).

[b14] Cassabois G., Valvin P. & Gil B. Hexagonal boron nitride is an indirect bandgap semiconductor. Nat. Photonics 10, 262–267 (2016).

[b15] Li X. . Large-area synthesis of high-quality and uniform graphene films on copper foils. Science (80-.). 324, 1312–1314 (2009). 10.1126/science.1171245 19423775

[b16] Zhao L. . Local Atomic and Electronic Structure of Boron Chemical Doping in Monolayer Graphene. Nano Lett. 13, 4659–4665 (2013). 24032458 10.1021/nl401781d

[b17] Kidambi P. R. . *In situ* observations during chemical vapor deposition of hexagonal boron nitride on polycrystalline copper. Chem. Mater. 26, 6380–6392 (2014). 25673919 10.1021/cm502603n PMC4311958

[b18] Kim K. K. . Synthesis of monolayer hexagonal boron nitride on Cu foil using chemical vapor deposition. Nano Lett. 12, 161–166 (2012). 22111957 10.1021/nl203249a

[b19] Tay R. Y. . Growth of large single-crystalline two-dimensional boron nitride hexagons on electropolished copper. Nano Lett. 14, 839–846 (2014). 24447201 10.1021/nl404207f

[b20] Platzman I., Brener R., Haick H. & Tannenbaum R. Oxidation of Polycrystalline Copper Thin Films at Ambient Conditions. J. Phys. Chem. C 112, 1101–1108 (2008).

[b21] Barr T. L. An ESCA study of the termination of the passivation of elemental metals. J. Phys. Chem. 82, 1801–1810 (1978).

[b22] Barr T. L. ESCA studies of naturally passivated metal foils. J. Vac. Sci. Technol. 14, 660 (1977).

[b23] Iijima J. . Native oxidation of ultra high purity Cu bulk and thin films. Appl. Surf. Sci. 253, 2825–2829 (2006).

[b24] Zhang Y. H. . The distribution of wrinkles and their effects on the oxidation resistance of chemical vapor deposition graphene. Carbon N. Y. 70, 81–86 (2014).

[b25] Yin X. . Evolution of the Raman spectrum of graphene grown on copper upon oxidation of the substrate. Nano Res. 7, 1613–1622 (2014).

[b26] O’Reilly M. . Investigation of the oxidation behaviour of thin film and bulk copper. Appl. Surf. Sci. 91, 152–156 (1995).

[b27] Kaniyoor A., Baby T. T. & Ramaprabhu S. Graphene synthesis via hydrogen induced low temperature exfoliation of graphite oxide. J. Mater. Chem. 20, 8467 (2010).

[b28] Poulston S., Parlett P. M. & Stone P. Sourface Oxidation and Reduction of CuO and Cu_2_O studied Using XPS and XAES. Surf. Interface Anal. 24, 811–820 (1996).

[b29] Moulder J. F., Stickle W. F., Sobol P. E. & Bomben K. Handbook of X-ray Photoelectron Spectroscopy. (Perkin-Elmer Corporation, 1992).

[b30] Duong D. L. . Probing graphene grain boundaries with optical microscopy. Nature 490, 235–239 (2012). 23034653 10.1038/nature11562

[b31] Gibb A. L. . Atomic resolution imaging of grain boundary defects in monolayer chemical vapor deposition-grown hexagonal boron nitride. J. Am. Chem. Soc. 135, 6758–6761 (2013). 23550733 10.1021/ja400637n

[b32] Wang C. . Growth of millimeter-size single crystal graphene on Cu foils by circumfluence chemical vapor deposition. Sci. Rep. 4, 4537 (2014). 24686949 10.1038/srep04537 PMC3971397

[b33] Hao Y. . The role of surface oxygen in the growth of large single-crystal graphene on copper. Science 342, 720–3 (2013). 24158906 10.1126/science.1243879

[b34] Zhou H. . Chemical vapour deposition growth of large single crystals of monolayer and bilayer graphene. Nat. Commun. 4, 2096 (2013). 23803650 10.1038/ncomms3096

[b35] Luo B. . Copper Oxidation through Nucleation Sites of Chemical Vapor Deposited Graphene. Chem. Mater. 28, 3789–3795 (2016).

[b36] Gibb A. L. . Atomic Resolution Imaging of Grain Boundary Defects in Monolayer Chemical Vapor Deposition-Grown Hexagonal Boron Nitride. J. Am. Chem. Soc. 135, 6758–6761 (2013). 23550733 10.1021/ja400637n

[b37] Zhu Y., Mimura K. & Isshiki M. Oxidation Mechanism of Copper at 623–1073 K. Mater. Trans. 43, 2173–2176 (2002).

