# Supplementary material for: Real-time oxide evolution of copper protected by graphene and boron nitride barriers
Source: Sci Rep. 2017 Jan 9;7:39770. doi: 10.1038/srep39770 (PMC5220376; doi:10.1038/srep39770)
Supplement: Supplementary Information [file srep39770-s1.pdf]

SUPPLEMENTARY INFORMATION

Real-time oxide evolution of copper protected by graphene and boron nitride barriers

*M. Galbiati, A. C. Stoot, D. M. A. Mackenzie, P. Bøggild, L. Camilli**

Department of Micro- and Nanotechnology, DK-2800 Kgs. Lyngby, Denmark

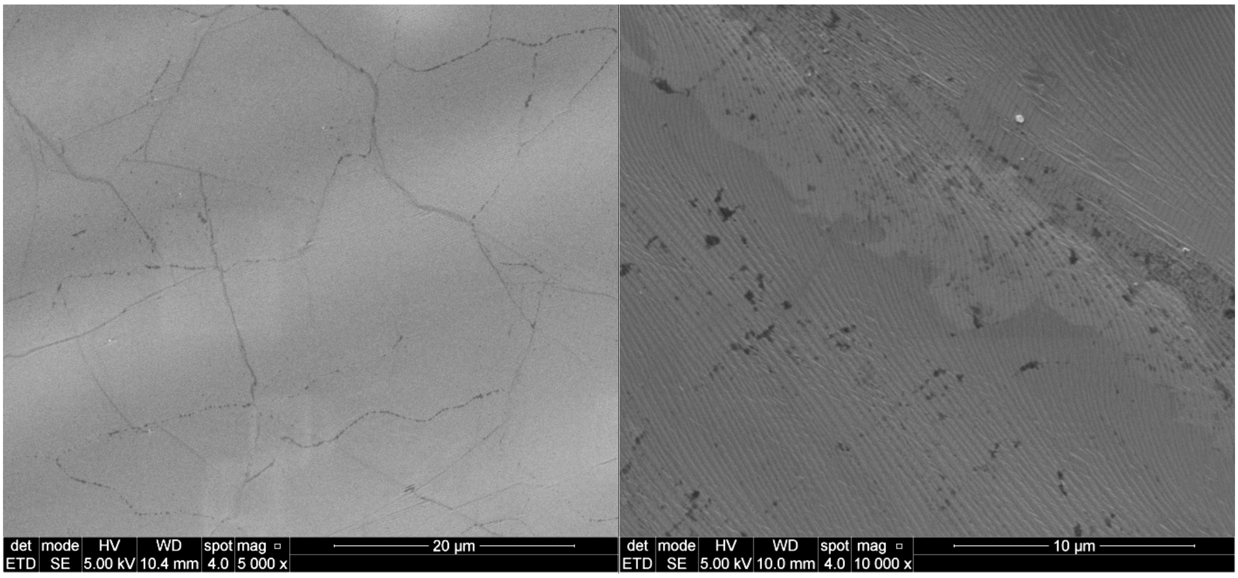

Fig. S1. SEM images of a sample of graphene grown on copper. Before the SEM experiment, the sample has been heated at 180°C for less than 5 minutes in air on a hotplate in order to make the grain boundaries of graphene more visible. No wrinkle can be observed in the scanned area, while the darker lines are to be assigned to oxidized part on the copper surface as a result of oxygen passing through the boundaries between graphene grains. The overall copper surface is still clean and not-oxidized, as one can realize by looking at the steps and terraces in the zoom-in image at right.

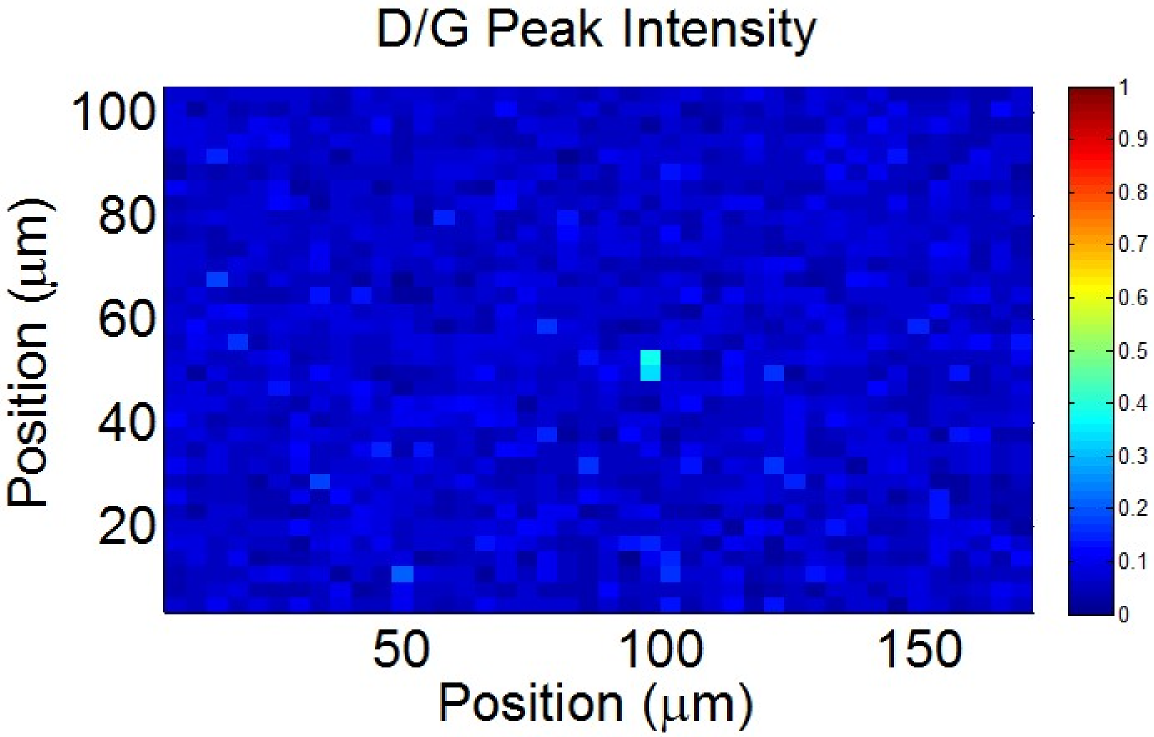

Fig. S2. D/G Raman map of an area 102x168μm^2 of as-grown graphene on copper. Map parameters: X step size, 4μm; Y step size, 3μm.

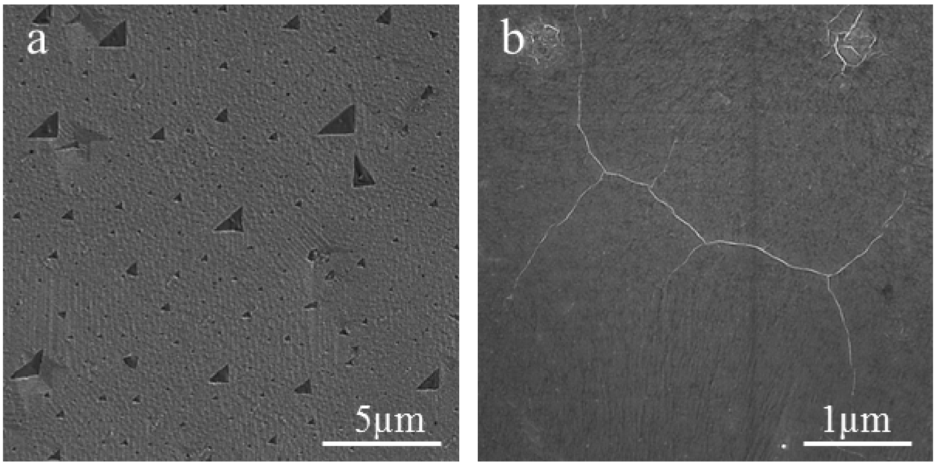

Fig. S3 SEM images of (a) hBN-coated sample prepared with a growth time of only 5 minutes, in order to show the high density of nucleation within our growth procedure. In order to make the hBN nuclei more visible, the copper surface has been oxidized at 150°C before taking the SEM pictures. The black triangles are the hBN nuclei. (b) hBN-coated sample showing wrinkles with elongated morphology, like the one running at the centre of the picture, and 'flower-shaped' ones, visible at the top left and top right corners of the picture.

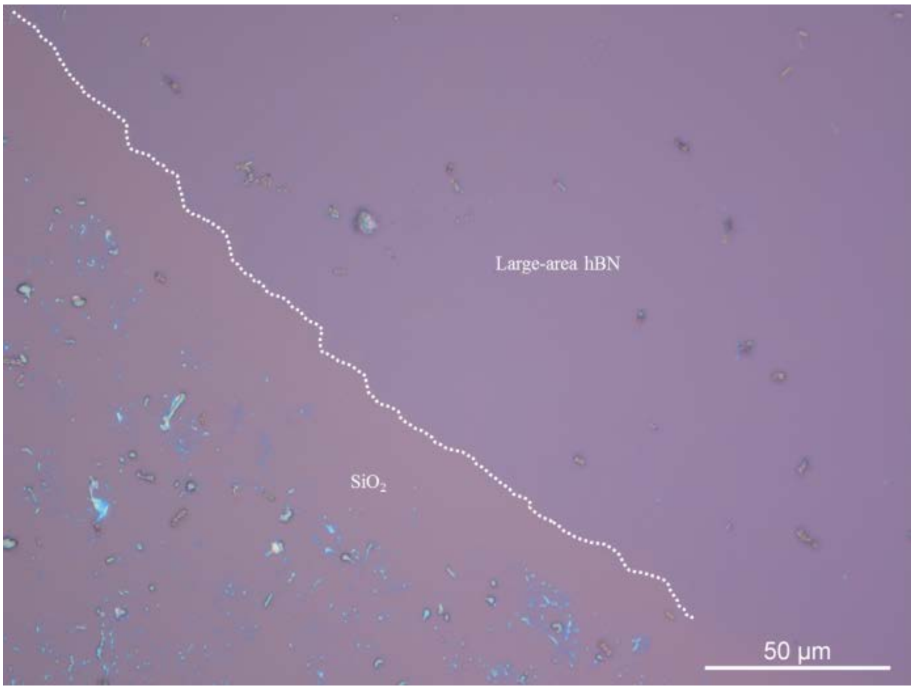

Fig. S4. Optical image of the hBN layer transferred on a SiO_2/Si substrate after growth. The white dashed line markes the edge of the transferred hBN sheet.

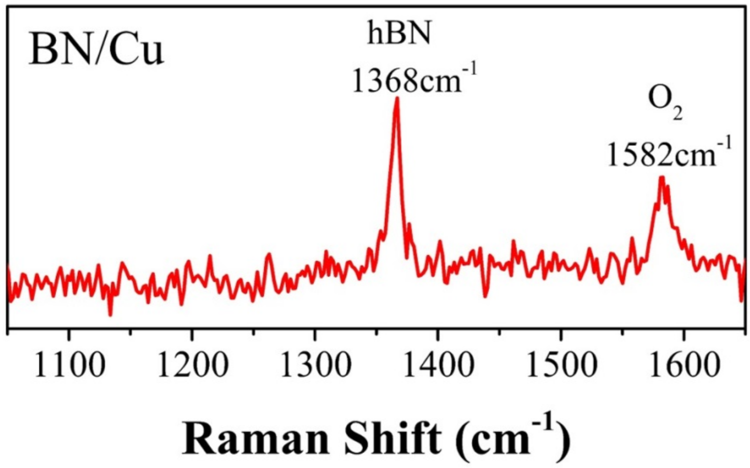

Fig. S5. Raman spectrum of as-grown hBN film on copper.

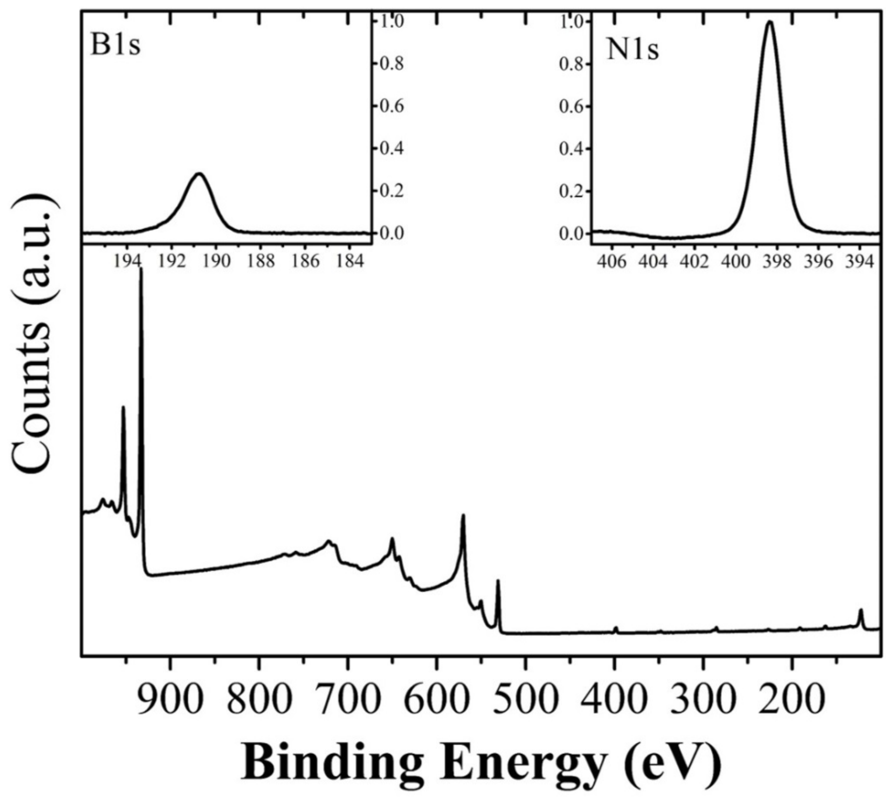

Fig S6. XPS (Al kα) survey spectrum of as-grown hBN on copper. The B1s and N1s peaks, hardly visible in the full energy range survey, are blown-up in the insets.

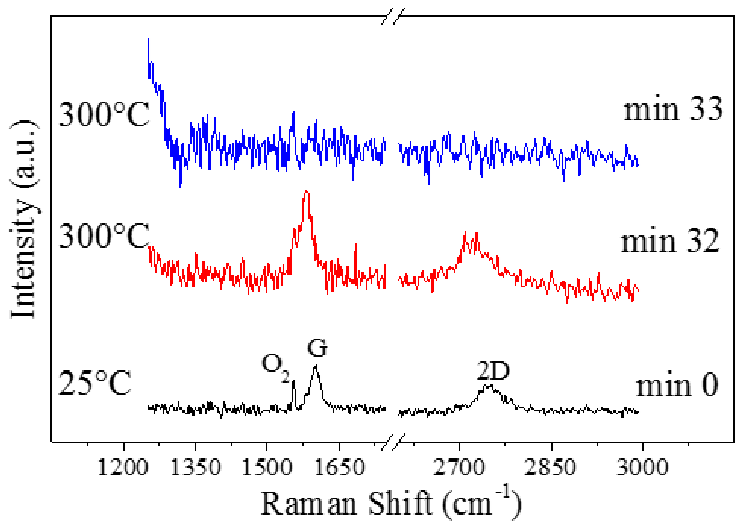

Fig. S7 Raman spectra collected before the variable temperature experiment (black curve, min 0), at 300 °C after 32 minutes (red curve) and (stiil at 300°C) after 33 minutes (blue curve). At minute 33, graphene oxidized upon reaction with ambient oxygen and thus neither G nor 2D mode signals are visible in the Raman spectrum.

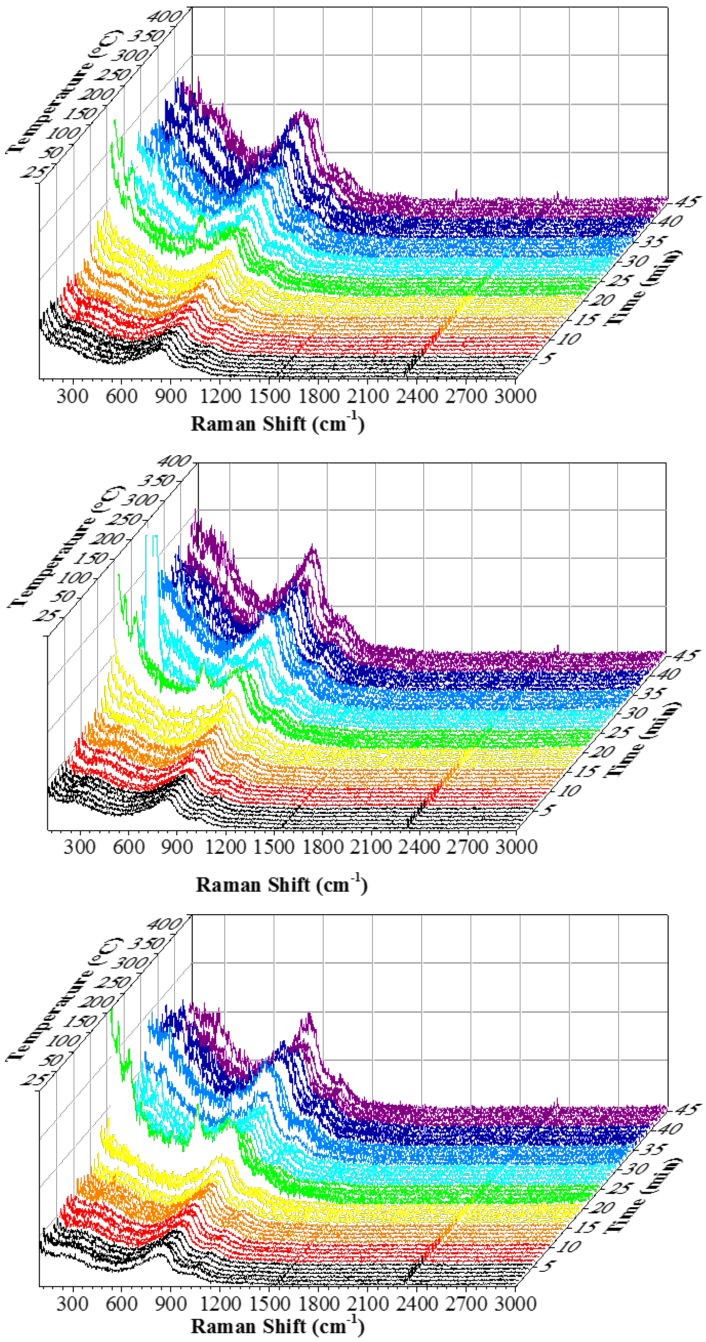

Fig. S8 Evolution of Raman spectra for three hBN–coated samples during the variable temperature experiment to 400°C.

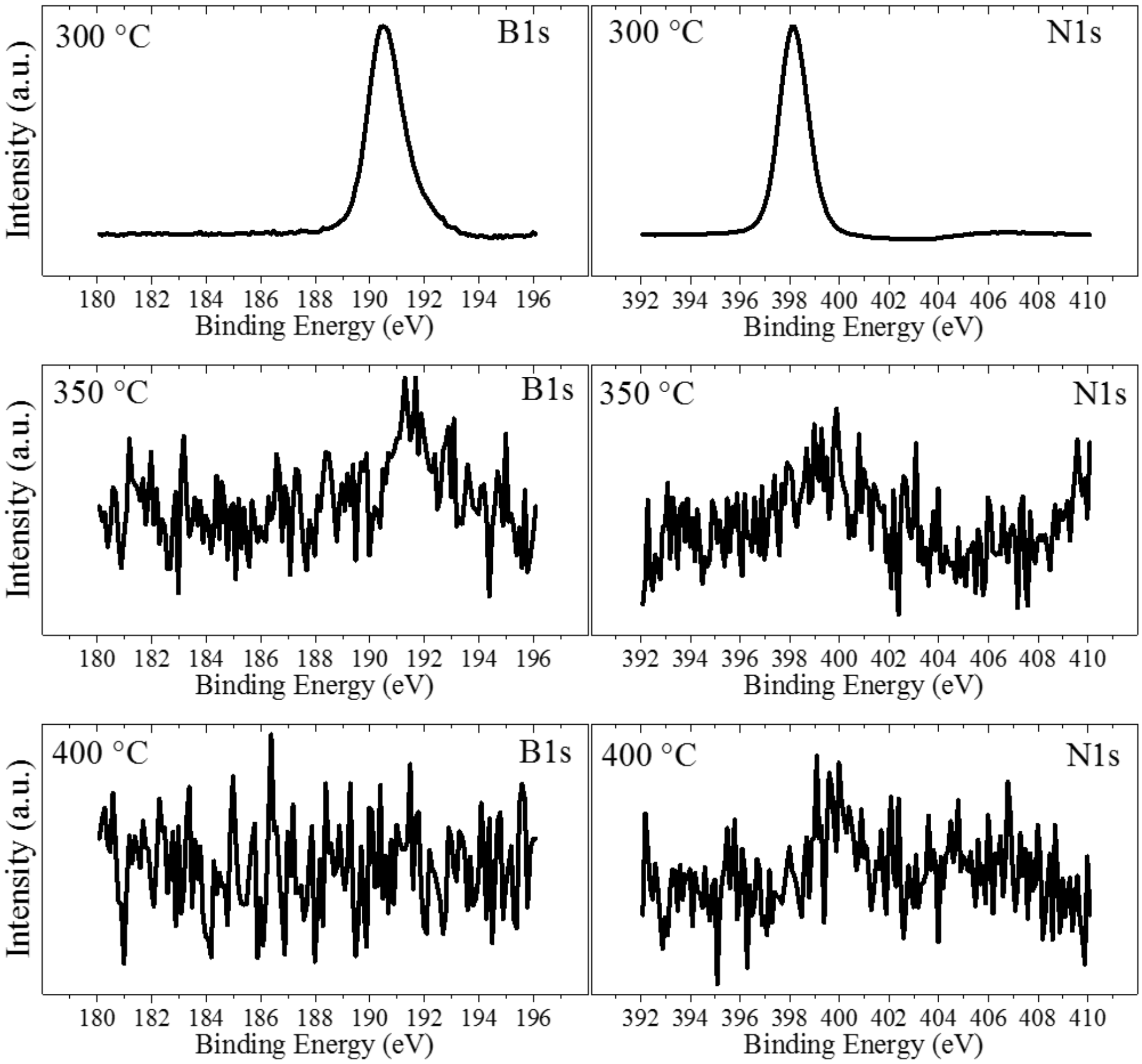

Fig. S9 XPS spectra of B1s and N1s for hBN-coated sample after being heated to 300°C (top panel), 350°C (centre panel) and 400 °C (bottom panel). At 300°C there is still hBN on the surface of the sample, while at 350°C both N and B signals drop almost at the noise level. B1s is shifted towards higher binding energy, this indicating the formation of boron oxide. At 400°C, B signal is not detectable anymore.

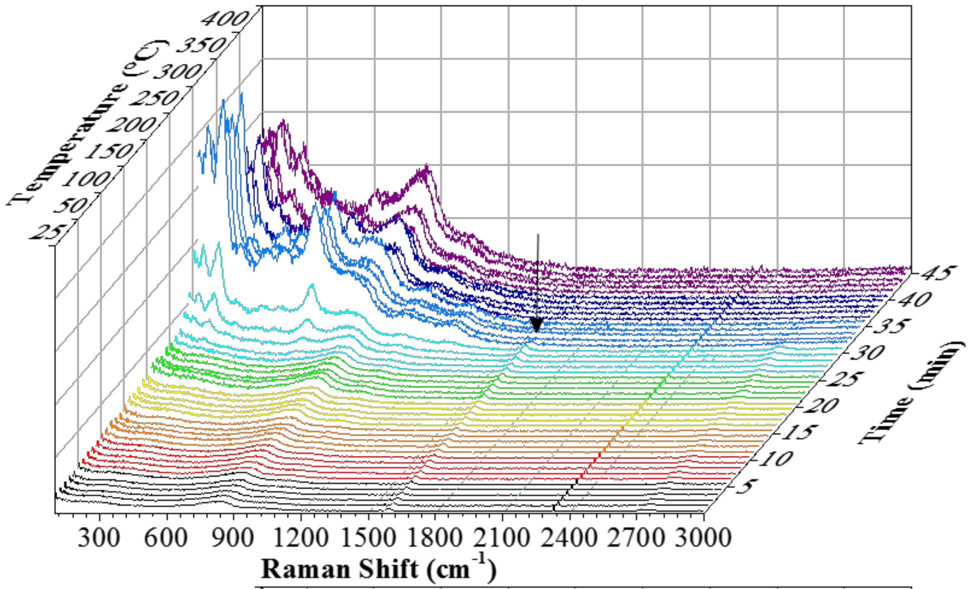
Temperature (°C)
25 50 100 150 200 250 300 350 400
Time (min)
5 10 15 20 25 30 35 40 45
300 600 900 1200 1500 1800 2100 2400 2700 3000
Raman Shift (cm-1)

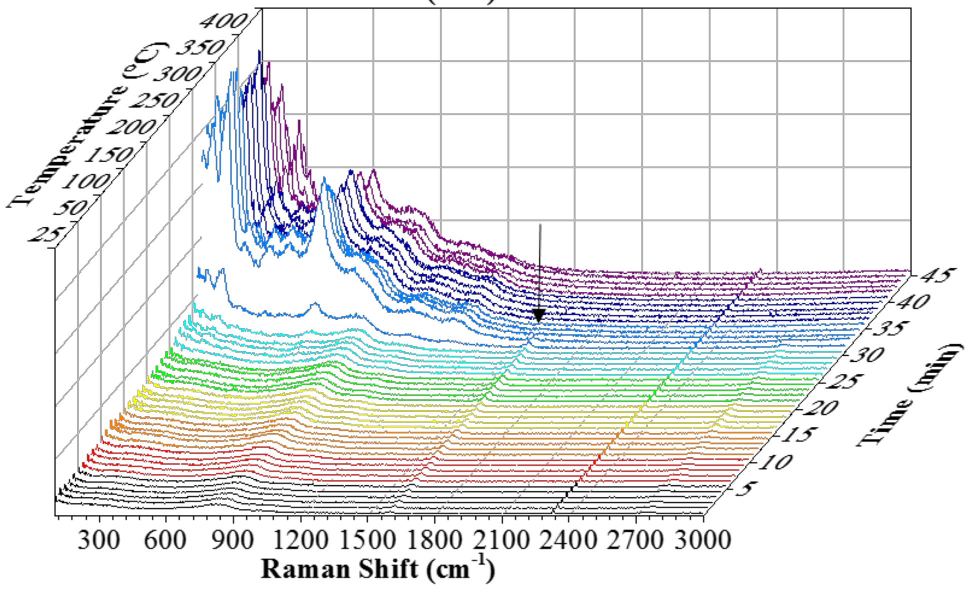
Temperature (°C)
25 50 100 150 200 250 300 350 400
Time (min)
5 10 15 20 25 30 35 40 45
300 600 900 1200 1500 1800 2100 2400 2700 3000
Raman Shift (cm-1)

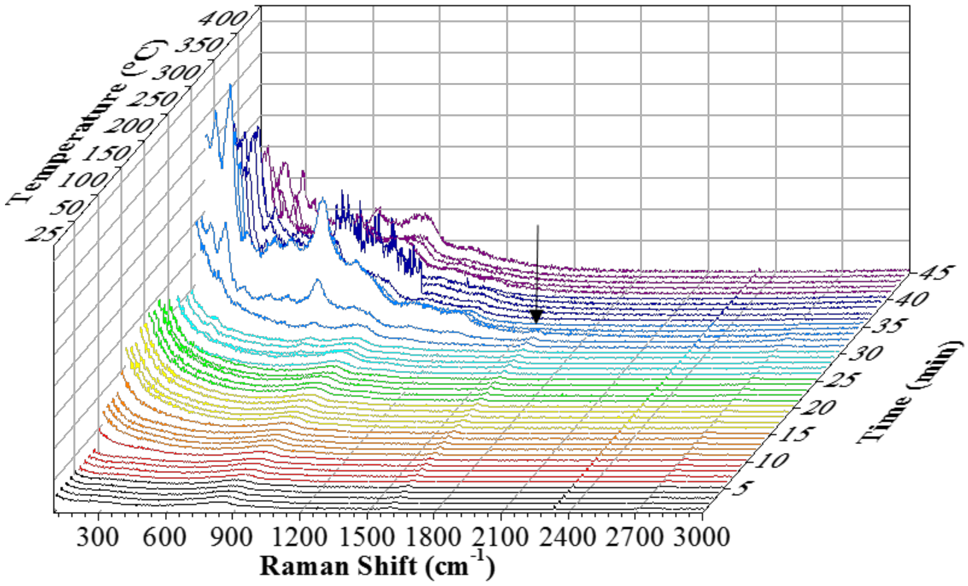
Temperature (°C)
25 50 100 150 200 250 300 350 400
Time (min)
5 10 15 20 25 30 35 40 45
300 600 900 1200 1500 1800 2100 2400 2700 3000
Raman Shift (cm-1)

Fig S10 Evolution of Raman spectra for three G–coated samples during the variable temperature experiment. The oxide peaks rapidly increase their intensities when the graphene is etched upon reacting with ambient oxygen (indicated by the black arrow).

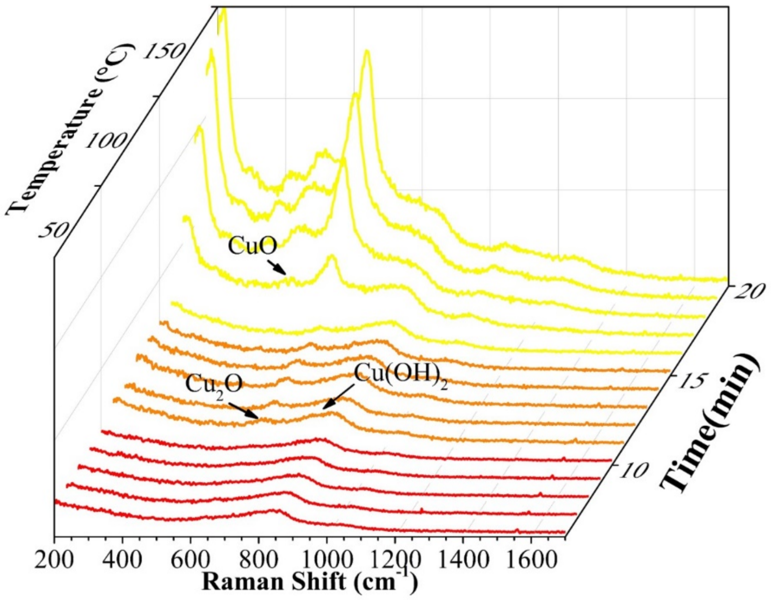

Fig. S11 Evolution of Raman spectra for bare Cu sample during the variable temperature experiment between 50°C and 150°C. The figure points out the earlier formation of Cu2O and $Cu(OH)_2$ at 100°C respect to CuO which is formed later at 150°C.

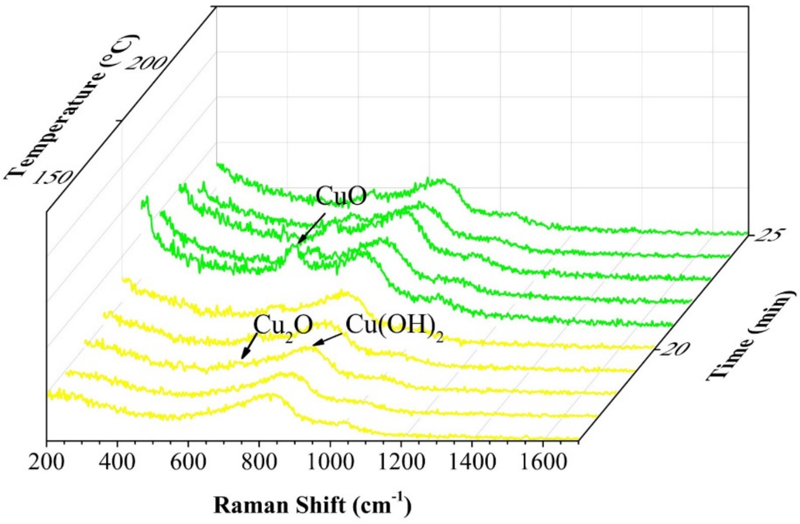

S. 12 Evolution of Raman spectra for hBN–coated sample during the variable temperature experiment between 150°C and 200°C. The figure highlights the earlier formation of Cu2O and Cu(OH)2 at 150°C respect to CuO which is formed later at 200°C.

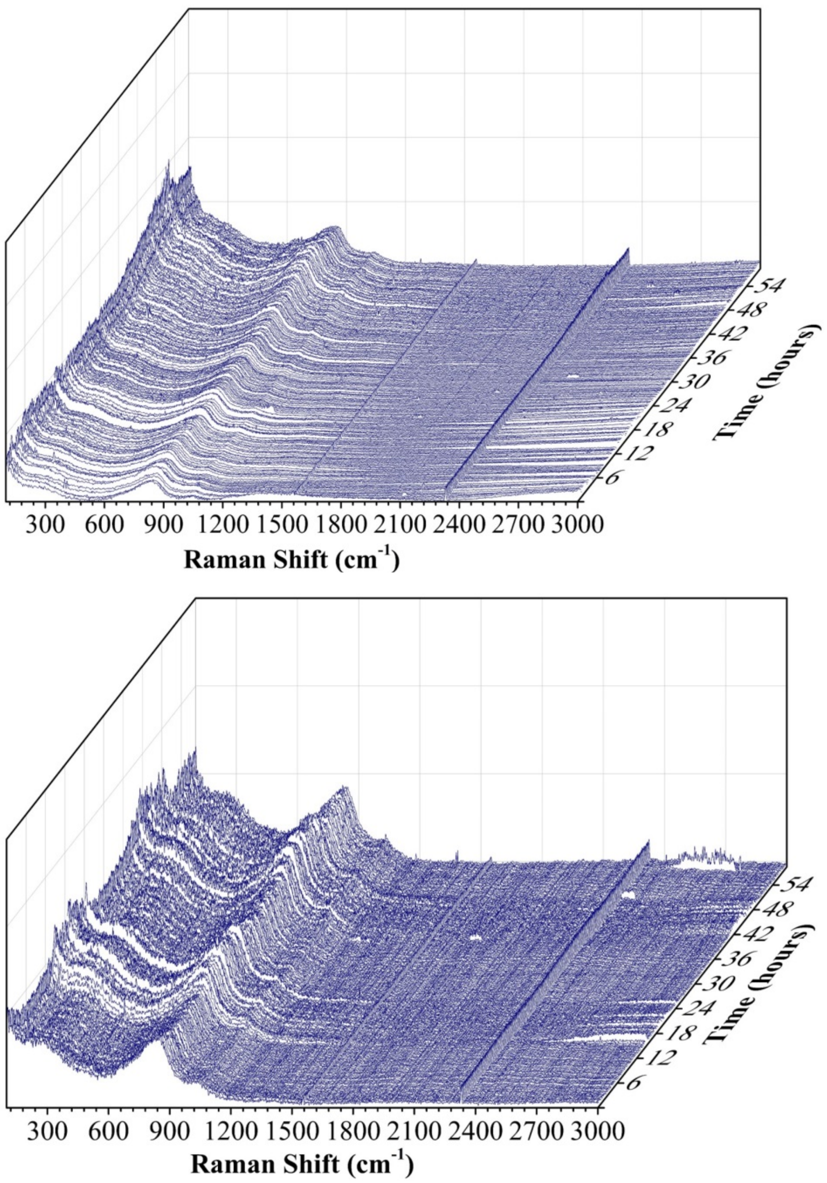

Fig. S13 Evolution of Raman spectra for two hBN–coated samples during the isothermal oxidation experiment. The characteristic hBN peak at ~1368cm^{-1} is not visible here because of the small collection time used to follow in *real-time* the oxidation of the copper surface during the experiment.

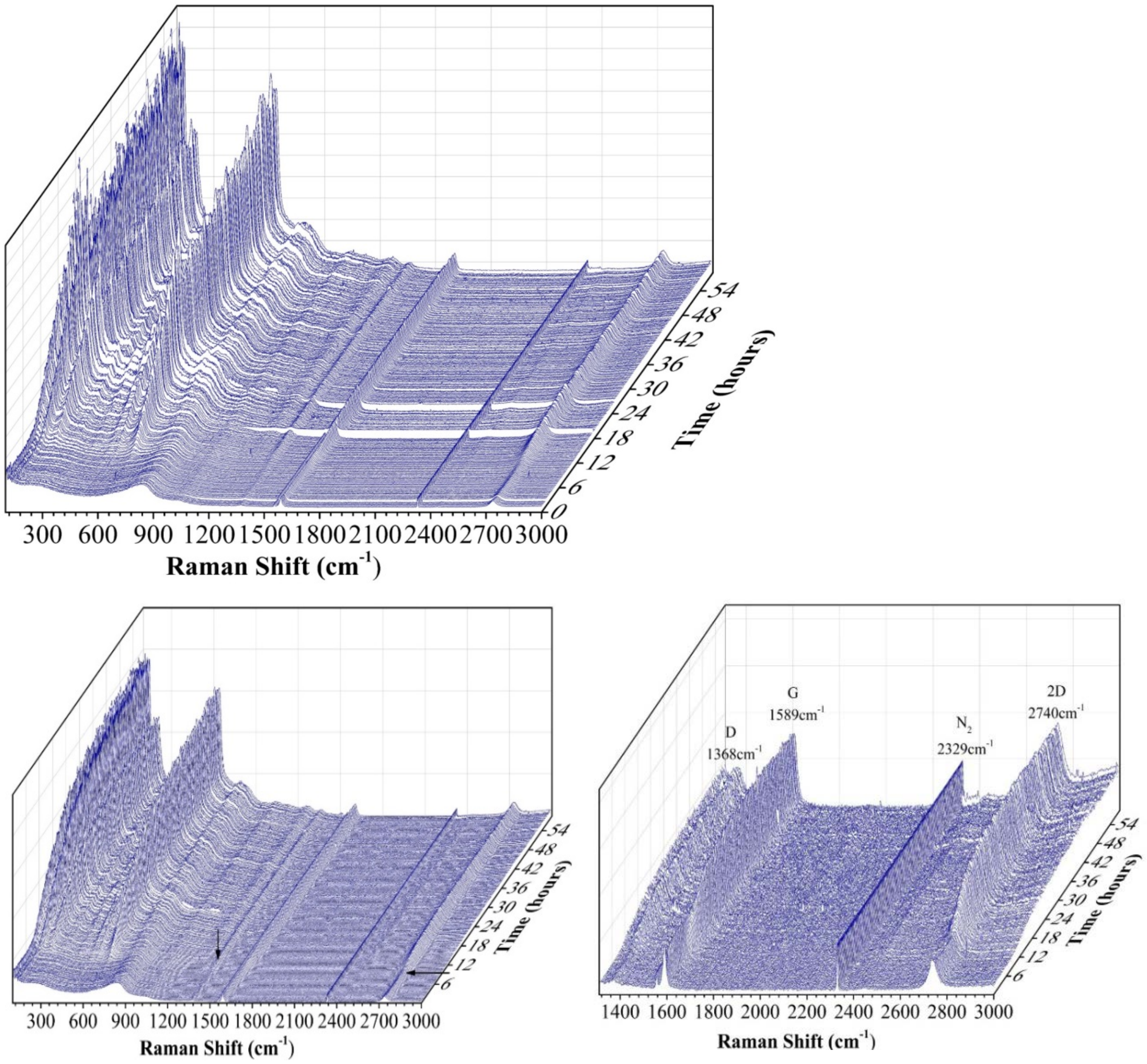

Fig. S14 Evolution of Raman spectra for two G–coated samples (upper panel and first spectrum in lower row) during the isothermal oxidation experiment. The second spectrum (lower raw, spectrum at left) has been also plotted in the range 1320-3000cm^{-1} in order to better appreciate the evolution of graphene D, G and 2D peaks during the isothermal experiment (right panel in the lower row). After 9h (see black, horizontal arrow), graphene 2D (at 2740cm^{-1}) and G (1589cm^{-1}) mode become more intense (while the nitrogen peak at 2329cm^{-1} (i.e., N_2) remains constant) and, at the same time, undergo a red shift of 5cm^{-1} and a blue shift of 23cm^{-1}, respectively. This is due the intercalation of a layer of oxygen, which acts like a spacer between the graphene layer and the Cu surface. Graphene is now decoupled from the Cu surface. The D peak (1368cm^{-1}) appears around 12h (see black, vertical arrow), thus few hours after the beginning of the oxidation. The development of D-peak is suggested to be due to mechanical stresses induced by the oxidation – and consequent crystal modification – of the copper surface[2].

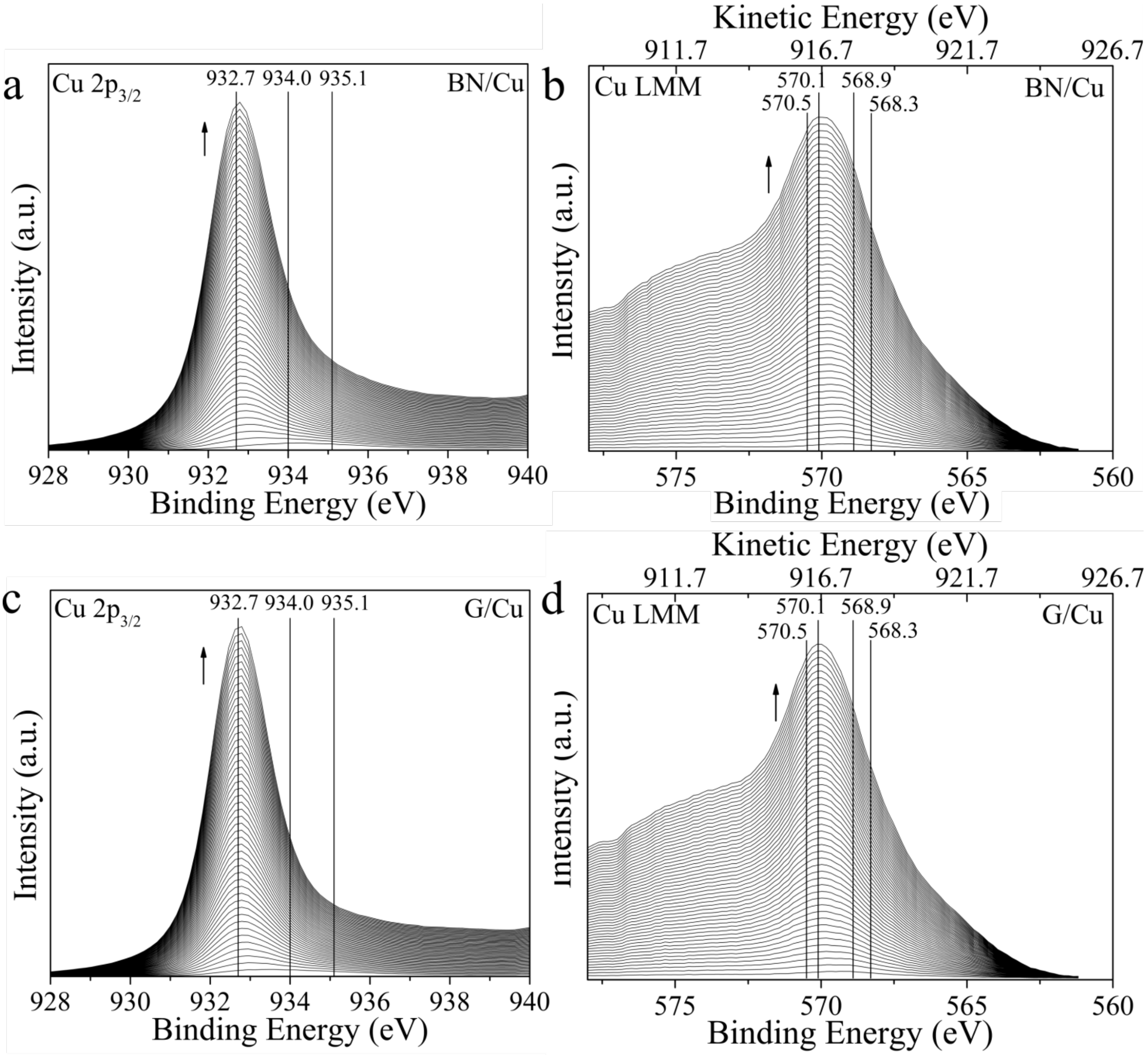

Fig. S15 XPS and X-ray Auger Spectroscopy (XAES) depth-profile of BN-coated (*a* and *b*)and G-coated (*c* and *d*) copper samples after variable temperature experiment at 400°C. Al kα is the excitation source, while an Ar^+ ion gun is used for sputtering; ~2.5nm of the sample are etched away at each sputtering cycle. In each graph several curves are shown; from the one collected on the as-loaded sample (the bottom curve) to the one after the last sputtering cycle (the top curve), as indicated by the arrows.

Both the samples show a similar oxide composition: at the surface (lower curves), the main oxide is CuO (934.0eV in XPS – left side – and 568.9eV in XAES – right side), which gradually disappears within the first 25-35nm from the surface. From this depth, the main oxide becomes Cu_2O (932.7eV in XPS – left side – and 570.1eV in XAES – right side).

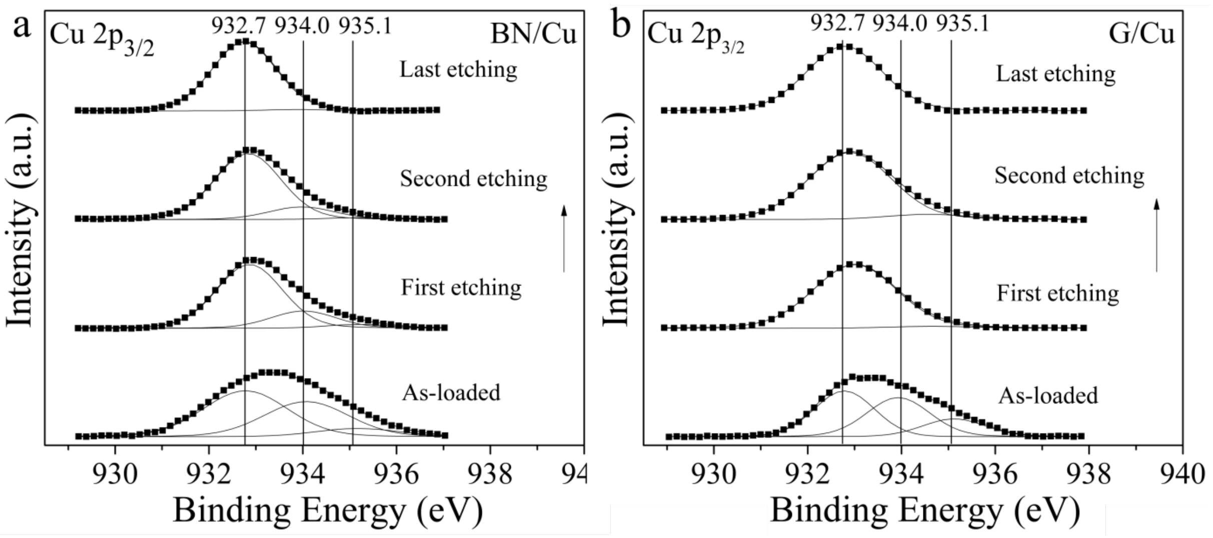

Fig. S16. Extracted from the XPS data in Fig. S15. Here, for the sake of clarity, only the XPS curves for the samples as-loaded, after the first sputtering cycle, the second and the last one are reported.

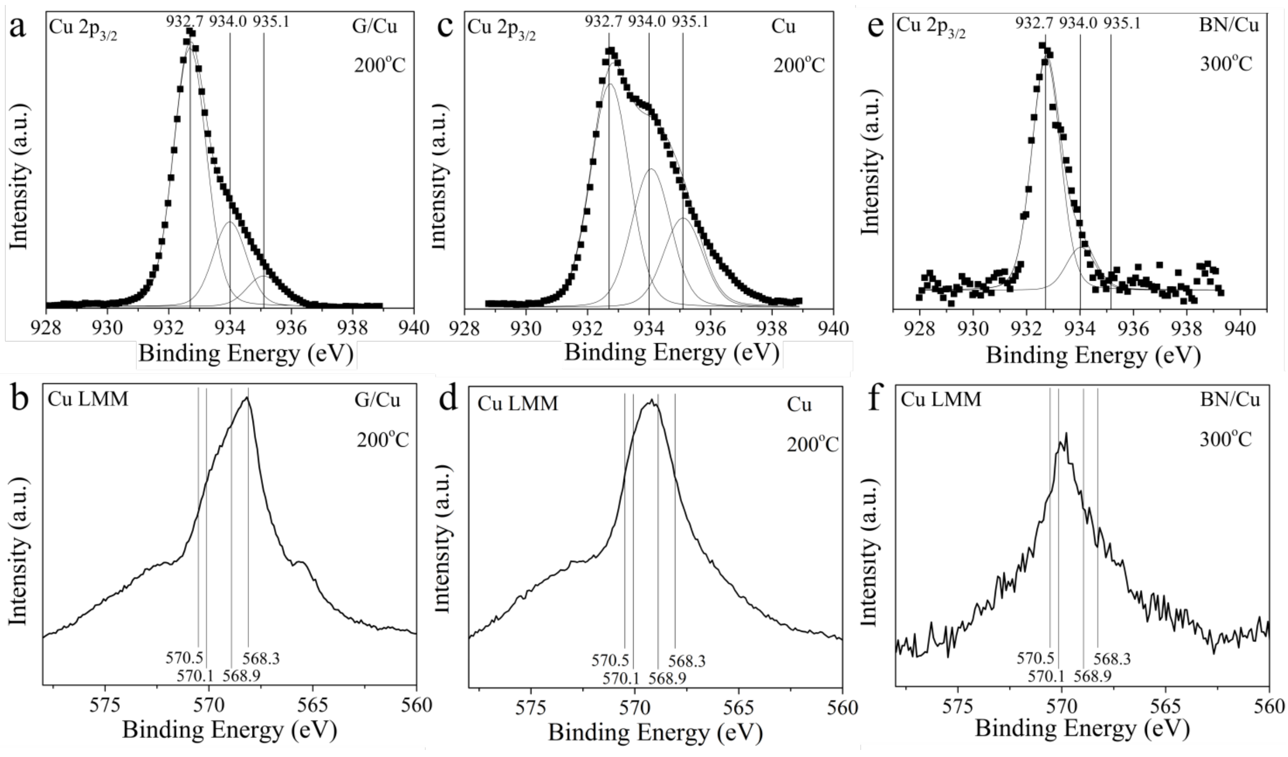

Fig. S17. XPS (upper row)and XAES (lower row) of graphene-coated, bare and BN-coated copper samples after the variable temperature experiment at 200°C for graphene-coated and bare copper samples and at 300°C for BN-coated one. At 200°C, the copper surface protected by the graphene coating shows sign of oxidation (**a**, see the two components at 934eV and 935.1eV, representing CuO and $Cu(OH)_2$ respectively), but the Cu areas are still dominating (**b**, the Auger line is peaked around 568.3eV). At the same temperature, the surface of the bare copper sample is more oxidized (the intensity of the peaks at 934eV and 935.2eV in **c** are higher and the relative Auger peak in **d** is centered in the range 568.9 - 570.1eV, representing respectively CuO and Cu_2O. At 300°C – the limit temperature for BN coatings, before reacting with ambient oxygen at 350°C, the copper surface appears oxidized, with the main oxide being Cu_2O (932.7eV in **e** and 570.1eV in **f**). Nevertheless, the presence of the non-protective CuO is considerably lower than in the case of bare copper oxidized at 200°C (**c** and **d**).

REFERENCES

1. Moulder J. F., et al. 1992 Handbook of X-ray Photoelectron Spectroscopy 2nd edn.
2. Zhou F, et al. 2013 ACS Nano, 7, 6939-6947
